# IS*26*-mediated circular *bla_KPC_* transposon in ST2483 *Pseudomonas aeruginosa*

**DOI:** 10.1128/aac.00582-26

**Published:** 2026-08-24

**Authors:** Mingxiao Chen, Qingqing Zhi, Miaoshan Luo, Meifang Lin, Canbo Fan, Zhuoyan Zhong, Tingting Deng, Jingyi Zhang, Qiwen Xiao, Jianqiang Zhu, Yanfen Lin, Minling Wang, Xiaobing Duan, Cong Shen, Qiang Zhou

**Affiliations:** 1 Clinical Laboratory Medicine Department, The Second Affiliated Hospital, Guangzhou Medical University, Guangzhou, China; 2 College of Agriculture and Biology, Zhongkai University of Agriculture and Engineering, Guangzhou, China; 3 School of Basic Medicine, Guangzhou Medical University 26468, Guangzhou, China; 4 Clinical Laboratory Medicine Department, The Third Affiliated Hospital, Guangzhou Medical University, Guangzhou, China; 5 The Second Clinical Medical College, Guangzhou University of Chinese Medicine, State Key Laboratory of Traditional Chinese Medicine Syndrome, Guangdong Provincial Hospital of Chinese Medicine, Guangzhou, China; 6 State Key Laboratory of Dampness Syndrome of Chinese Medicine/Clinical Laboratory, Guangdong Provincial Hospital of Chinese Medicine, Guangzhou, China; Universita degli Studi di Roma La Sapienza, Rome, Italy

**Keywords:** carbapenem-resistant *Pseudomonas aeruginosa *(CRPA), ST2483, *bla*_KPC_, IS*26-*mediated circular transposon, outer membrane vesicles (OMVs)

## Abstract

The global spread of carbapenem-resistant *Pseudomonas aeruginosa* (CRPA) poses a critical clinical threat, with *bla*_KPC_ as a key mobile carbapenem resistance gene. Here, we investigated a novel *bla*_KPC-2_ genetic element and its transmission in clinical ST2483 isolate PA100 via whole-genome sequencing, molecular, and phenotypic testing, and vesicle analysis. A unique 4,746-bp IS*26*-flanked structure inserted at the chromosomal *InaA/pncB2* locus was confirmed, which forms a circular transposition intermediate through IS*26* recombination. PA100 exhibited ~40-fold higher *bla*_KPC-2_ copy number and ~25-fold higher mRNA expression vs chromosomal *bla*_KPC-2_ strain PA3; meropenem exposure further elevated its transcription by nearly 70-fold, far exceeding controls. PA100 showed an extensively drug-resistant phenotype with high-level carbapenem resistance, while remaining susceptible to ceftazidime-avibactam and cefiderocol. No classical conjugation or transformation was observed, while intact outer membrane vesicles (OMVs) from PA100 carried *bla*_KPC-2_, supporting a non-plasmid, vesicle-associated route for transient delivery of the resistance gene under the tested conditions. The circular transposon remained genetically stable after 10 generations of passaging in PA100; however, stable maintenance in the PAO1 recipient was not detected after passage. In summary, we first report an IS*26*-driven circular *bla*_KPC-2_ transposon in ST2483 *P. aeruginosa*, conferring robust overexpression and stable inheritance in the donor strain, with OMVs potentially facilitating its horizontal spread, although stable maintenance in recipient cells was not demonstrated under the current assay conditions. These data advance understanding of *bla*_KPC_ dissemination in CRPA and highlight clinical surveillance priorities for rare high-risk sequence types.

## INTRODUCTION

Carbapenem-resistant *Pseudomonas aeruginosa* (CRPA), designated as a priority pathogen by the World Health Organization, presents a formidable challenge to global health, characterized by limited therapeutic options and a rising incidence rate (1–3). The *bla*_KPC_ gene, which encodes a carbapenemase, stands as a pivotal determinant of carbapenem resistance in CRPA (2, 4). The horizontal transfer of *bla*_KPC_ to *P. aeruginosa* has significantly complicated clinical management, thereby underscoring the imperative need for a more profound comprehension of its dissemination mechanisms (5, 6). However, the extent to which carbapenemase genes carried by *P. aeruginosa* contribute to the spread of carbapenem resistance among Gram-negative bacteria remains to be elucidated.

The clonal diversity of *P. aeruginosa* complicates CRPA epidemiology. While certain sequence types (STs) such as ST235 and ST463 dominate in Europe and China, respectively (6–9), the rare ST2483 lineage harboring *bla*_KPC-2_ underscores the emergence of new transmission clones. This genetic and geographic heterogeneity underscores the need for targeted surveillance of both bacterial genotypes and mobile genetic elements (MGEs) to predict resistance spread (10, 11).

MGEs, particularly insertion sequences (IS), are instrumental in mobilizing antibiotic resistance genes (12). IS*26*, a highly active IS, is capable of recruiting individual genes or groups of genes into the mobile gene pool and facilitating their sustained dissemination to novel genomic loci through the formation of pseudo-compound transposons (PCTs) (13, 14). These PCTs can be further mobilized by IS*26*, enhancing the spread of resistance genes (12, 15). Additionally, IS*26* can upregulate the expression of adjacent potential resistance genes (14, 16–18). The module IS*26*-∆*tnpA_Tn3_-ISKpn27-bla_KPC-2_-ISKpn6* represents a recurrent *bla*_KPC-2_ context in *Enterobacterales*, and acquisition of an additional flanking IS*26* can generate a double-IS*26*-bounded structure with potential to form circular intermediates. Despite these known functions, the precise mechanisms by which *bla*_KPC-2_ is disseminated in *P. aeruginosa* remain poorly understood.

Horizontal gene transfer of antimicrobial resistance genes represents a critical driver of bacterial resistance. Although traditional mechanisms, including conjugation, transformation, and transduction, are well documented, these routes do not explain all observed movement of resistance determinants, particularly when a transferable plasmid or stable transformant is not detected (19–21). Bacterial outer membrane vesicles (OMVs) are lipid-bilayer-enclosed nanoparticles (20–200 nm in diameter) that facilitate gene transfer, bacterial metabolism, and intercellular communication (19, 20). OMVs encapsulate nucleic acids, including antibiotic resistance genes such as *bla*_NDM-1_, *bla*_OXA-48_, and *bla*_CTX-M-15_(22–25), thereby protecting them from external nucleases and facilitating their long-distance transfer to recipient strains. However, the extent to which OMVs mediate the dissemination of *bla*_KPC-2_ in CRPA remains unexplored, underscoring the need for further investigation into this potential pathway of antibiotic resistance transfer.

In this study, we report the identification of a novel IS*26*-mediated circular transposon carrying *bla*_KPC-2_ in a rare ST2483 clinical CRPA strain PA100. This structure provides a compact *bla*_KPC-2_ module with the potential to generate circular intermediates. Our findings underscore the adaptability of MGEs in driving antimicrobial resistance and highlight the importance of detecting circular intermediates in CRPA surveillance.

## MATERIALS AND METHODS

### Isolation of PA100 strain and antimicrobial susceptibility testing

*P. aeruginosa* strain PA100 was isolated from a midstream urine sample collected on 28 February 2024 from a 90-year-old male patient diagnosed with urinary tract infection at the Second Affiliated Hospital of Guangzhou Medical University. The isolate was identified as *P. aeruginosa* by MALDI-TOF/MS (Bruker Daltonik GmbH, Bremen, Germany), with whole-genome sequencing (WGS) confirming this species identification. AST was performed and interpreted according to CLSI (2025) and EUCAST (2025) guidelines. Quality control strains (*E. coli* ATCC 25922, *K. pneumoniae* ATCC BAA-1705, and *E. coli* ATCC 35218) were included in each run, with the latter two specifically monitored for β-lactam/β-lactamase inhibitor combinations per CLSI standards (Table 1).

**TABLE 1:** The drug sensitivity results of the KPC strains: PA3, PA100, and PA328^*a*^

Antimicrobial agent	PA3	PA100	PA328	Method	
Cefazolin	<=6 mm, R	<=6 mm, R	<=6 mm, R	KB	
Cefuroxime	<=6 mm, R	<=6 mm, R	<=6 mm, R	KB	
Ceftazidime	>=64, R	>=64, R	>=64, R	MIC	
Ceftriaxone	<=6 mm, R	<=6 mm, R	<=6 mm, R	KB	
Cefepime	>=32, R	>=32, R	>=32, R	MIC	
Meropenem	>=16, R	>=16, R	>=16, R	MIC	
Meropenem	32, R	64, R	64, R	MIC	
Meropenem	<=6 mm, R	<=6 mm, R	<=6 mm, R	KB	
Imipenem	>=16, R	>=16, R	>=16, R	MIC	
Imipenem	32, R	64, R	64, R	MIC	
Imipenem	<=6 mm, R	<=6 mm, R	<=6 mm, R	KB	
Aztreonam	<=6 mm, R	<=6 mm, R	<=6 mm, R	KB	
Gentamicin	<=6 mm, R	<=6 mm, R	17 mm, R	KB	
Tobramycin	>=16, R	>=16, R	<=1, S	MIC	
Amikacin	<=2, S	<=2, S	<=2, S	MIC	
Ciprofloxacin	>=4, R	>=4, R	>=4, R	MIC	
Levofloxacin	>=8, R	>=8, R	>=8, R	MIC	
Nitrofurantoin	<=6 mm, R	<=6 mm, R	<=6 mm, R	KB	
Minocycline	<=6 mm, R	<=6 mm, R	<=6 mm, R	KB	
Tigecycline	<=6 mm, R	<=6 mm, R	10 mm, R	KB	
Colistin	2, S	2, S	<=0.5, S	MIC	
Chloroamphenicol	<=6 mm, R	<=6 mm, R	<=6 mm, R	KB	
Ampicillin-sulbactam	<=6 mm, R	<=6 mm, R	<=6 mm, R	KB	
Ticarcillin-clavulanic acid	>=128, R	>=128, R	>=128, R	MIC	
Piperacillin-tazobactam	12 mm, R	11 mm, R	9 mm, R	KB	
Cefoperazone-sulbactam	>=64, R	>=64, R	>=64, R	MIC	
Imipenem-relebactam	12.5 mm, R	13 mm, R	12 mm, R	KB	
Meropenem-vaborbactam	32/8, R	64/8, R	64/8, R	MIC	
Ceftazidime-avibactam	33 mm, S	24.5 mm, S	22 mm, S	KB	
Ceftazidime-avibactam	1/0.25, S	4/1, S	4/1, S	MIC	
Cefiderocol	<=0.125, S	0.125, S	0.125, S	MIC	

^*a*^ MIC values are expressed in μg/mL.

### Whole-genome sequencing and bioinformatic analyses

Whole-genome sequencing of PA100 was performed using paired-end sequencing with Illumina Novaseq (2 × 150 bp paired-end reads), and long sequencing was performed with PacBio Sequel II by Shanghai Biozeron Biotechnology Co., Ltd. (Shanghai, China). PacBio reads were assembled using Unicycler v0.4.8 and polished by Pilon v1.22 with default parameters. The assembled genome of *P. aeruginosa* strain PA100 was submitted to the NCBI GenBank database and annotated using the NCBI Prokaryotic Annotation Pipeline.

### Plasmid characterization, conjugation, and transformation experiment

To investigate the autonomous transfer ability of the *bla*_KPC-2_, *E. coli* J53 was employed as the recipient strain in conjugation experiments. Transconjugants were selected using LB agar plates (Huankai Biology, China) supplemented with 200 mg/L NaN_3_ and 2 mg/L meropenem. Their identification as *E. coli* and the presence of the *bla*_KPC-2_ gene were confirmed through PCR and validated using MALDI-TOF/MS.

Circular transferable unit DNA was extracted using a commercial kit (Sangon Biotech, China) and analyzed by agarose gel electrophoresis and restriction enzyme digestion (*Eco*NI and *Pst*I). Sequencing was performed using the Sanger method, and sequences were assembled and analyzed using BioEdit software.

For transformation, 1 µg of purified DNA was mixed with 50 µL of electrocompetent PAO1 cells and electroporated using a Gene Pulser X cell device at 1.75 kV, 200 Ω, and 25 µF in 1-mm-gap cuvettes. Immediately after electroporation, cells were suspended in 1 mL of SOC medium and incubated at 37°C for 2 h with shaking to allow recovery and expression of antibiotic resistance genes. The cells were then plated on LB-meropenem agar to select for transformants. For heat-shock transformation, DNA was added to competent DH5α cells, heat-shocked, and recovered in LB SOC medium. Transformants were selected on LB-meropenem agar plates and confirmed by KPC detection PCR.

### Detection of the carbapenemase gene and its surrounding genetic environment

Antimicrobial resistance genes (ARGs) were identified using ResFinder v4.7.2, and the genomic location of *bla*_KPC_ was determined using the same platform. The geographic and host-range analysis used NCBI nucleotide BLAST searches of two query structures: a 4,746-bp one-flanking-IS*26* module (IS*26*-∆*tnpA_Tn3_-ISKpn27-bla_KPC-2_-ISKpn6*) and a 5,450-bp double-IS*26*-flanked module (IS*26*-∆*tnpA_Tn3_-ISKpn27-bla_KPC-2_-ISKpn6- IS26*). Hits were retained only when they showed 100% query coverage, 100% nucleotide identity, and the Max Score thresholds described in Results 3.1, followed by manual de-duplication at the isolate level before species, year, and geographic summaries were generated. For the strains included in this study, MLST was conducted using the MLST v2.0 online tool (https://cge.food.dtu.dk/services/MLST/). Plasmid incompatibility types were identified using PlasmidFinder v2.1 (26). Comparative genomic analyses of plasmid sequences were performed using BLAST Ring Image Generator (BRIG v0.95) (27). Insertion sequence (IS) elements in the genetic context of plasmid-borne *bla*_KPC_ were analyzed using ISfinder (28), and the genetic environment of *bla*_KPC_ was visualized using Easyfig v2.2.5 (29). Plasmid mobility was predicted using oriTfinder2 (30) by identifying the origin of transfer (*oriT*) and related components with default parameters.

### Quantification of *bla*_KPC-2_ DNA copies, analysis of mRNA, and protein expression levels

The carbapenemase gene *bla*_KPC-2_ in strain PA100 was detected by PCR with primers KPC-F and KPC-R (Table S1). The KPC enzyme was identified in the KPC-harboring CRPA strain PA100, with PA3 and PA328 serving as controls, utilizing a colloidal gold assay kit (Dynamiker Biotechnology, China). Total genomic DNA was extracted from the cultured PA100 using a genomic DNA extraction kit (Sangon Biotech, China). The concentration of extracted DNA was adjusted to 0.04 ng/µL, and copy numbers of the *bla*_KPC-2_ were quantified using a QX200 Droplet Digital PCR system (Bio-Rad).

Relative gene expression levels calculated from qPCR data were analyzed using the 2^−ΔΔCt method. Statistical significance was assessed via Student’s *t*-test for two-group comparisons, or one-way analysis of variance followed by Tukey’s post-hoc test for multiple group comparisons, with a threshold of *P* < 0.05 considered statistically significant. All PCR primers used in this study are listed in Table S1, including primers for the detection of the *bla*_KPC_ genetic context (between *InaA* and *pncB2*), inverse PCR primers targeting the circular structure characterization of the *bla*_KPC_ gene, and primers for Sanger sequencing of the corresponding PCR amplicons.

### Isolation, purification of OMVs, and nanoparticle tracking analysis

Briefly, a single colony grown on LB agar plate was inoculated into 5 mL of LB broth medium for 7 h culture. The next day, 5 mL of the 7-h bacterial culture was inoculated into 500 mL of LB medium, followed by incubation at 37°C and 180 rpm for 14–16 h. To isolate OMVs, centrifugations (5,000 *g*, 15 min, 4℃; 10,000 *g*, 30 min, 4℃) were adopted to remove bacterial, cellular debris and vesicles larger than 1 µm. The supernatant was then filtered via a 0.22 µm membrane filter (Merck Millipore, Germany). The crude OMVs were concentrated by ultra-centrifugation at 120,000 *g* for 70 min at 4℃ with an SW 70Ti rotor (centrifuge Optima XPN-100 Beckman Coulter, USA) and resuspended in 1 mL PBS (Beijing Solarbio Biotechnology Co., Ltd., China).

The crude OMVs isolated by ultra-centrifugation were further purified using OptiPrep (60% iodixanol; Merck Millipore, Germany) density gradient ultra-centrifugation. Briefly, 1 mL of crude OMVs was placed at the bottom of a 13.2 mL ultra-centrifuge tube. Twelve milliliters of OptiPrep density gradient solutions containing 30%, 20%, 10%, and 5% equal volume density gradients was consecutively added to the centrifuged tubes, and gradients were centrifuged at 100,000 *g* for 16 h at 4℃ with an SW 41Ti rotor. The OMVs layer was collected and washed twice with 10-fold PBS volume at 100,000 *g* for 70 min at 4℃, and the pellet, purified OMVs, was resuspended with 500 µL of the solution containing 10 mM HEPES and 200 mM NaCl (pH 7.4). Subsequently, the purified OMVs solution was filtered again via a 0.22 µm membrane filter and plated on a blood-agar plate to assure sterility. The external DNA and proteins were digested using 2 U DNase I (Takara, Japan) and 100 µg/mL Protease K (Roche, Switzerland) according to the manufacturer’s instructions. The digested OMVs were then kept at −80℃. Nanoparticles in the isolated OMV suspensions were analyzed using an NS-90 Plus instrument (Zhuhai OMEC Instrument Co., Ltd.) based on static light scattering (SLS), equipped with a 632.8-nm laser and an sCMOS camera. All samples were diluted with PBS to a final volume of 2 mL and injected into the sample chamber using sterile syringes, yielding a particle concentration of 10⁸ particles per mL in accordance with the manufacturer’s instructions.

### OMVs morphology characterization

The morphology of purified OMVs was observed by a transmission electron microscope (TEM, H7700, Hitachi, Ltd., Japan). The OMVs suspension was obtained by 100-fold dilution of purified OMVs in PBS, dropped on a copper grid with a support membrane for 5 min. Excess liquid was removed, and the OMVs were stained with 1% phosphotungstic acid for 2 min. OMVs morphology was obtained using the TEM at 80 kV. The diameter (Z-ave) of vesicles and the polymer dispersion index were determined using Dynamic Light Scattering (DLS) on a Zetasizer Nano-ZS (Malvern, UK). For DLS, OMVs were diluted 100-fold in PBS and measured in a clean quartz cuvette. All measurements were taken at 25℃, and each purification was tested in triplicates. The DLS data were processed by Malvern Panalytical (Malvern, UK) using Zeta sizer (V 7.11).

### Co-incubation of PA100-derived OMVs with PAO1^Rif^

Recipient *P. aeruginosa* PAO1^Rif^ was cultured in LB broth to logarithmic phase (OD600 = 0.6–0.8). The bacterial suspension was adjusted to 1 × 10^8^ CFU/mL. Purified PA100-derived OMVs (sterilized by 0.22 μm filtration and confirmed to be bacteria-free) were added to PAO1^Rif^ suspension at a final protein concentration of 50–100 μg/mL. The mixture was incubated at 37°C with shaking at 150 rpm for 6 h.

After co-incubation, the cultures were 10-fold serially diluted. Aliquots (100 μL) were spotted onto LB agar plates with meropenem (2 mg/L) and rifampicin (200 mg/L) for the selection of putative transformants, incubated at 37℃ for 24–48 h for total viable count. Single colonies grown on selective plates were subjected to colony PCR for the detection of *bla*_KPC_ and successive passages to obtain Passage 2 (P2) isolates. To calculate transfer efficiency, the total viable rifampicin-resistant recipient PAO1 cells were enumerated by spreading serial dilutions of the co-culture onto LB agar plates containing only rifampicin. The OMV-mediated transfer frequency was calculated as follows: transfer frequency = number of meropenem- and rifampicin-resistant transformants/total viable rifampicin-resistant recipient PAO1 cells.

## RESULTS

### Geographical distribution and host range of two distinct IS*26*-associated *bla*_KPC_ genetic contexts in bacterial isolates

**Fig 1:**
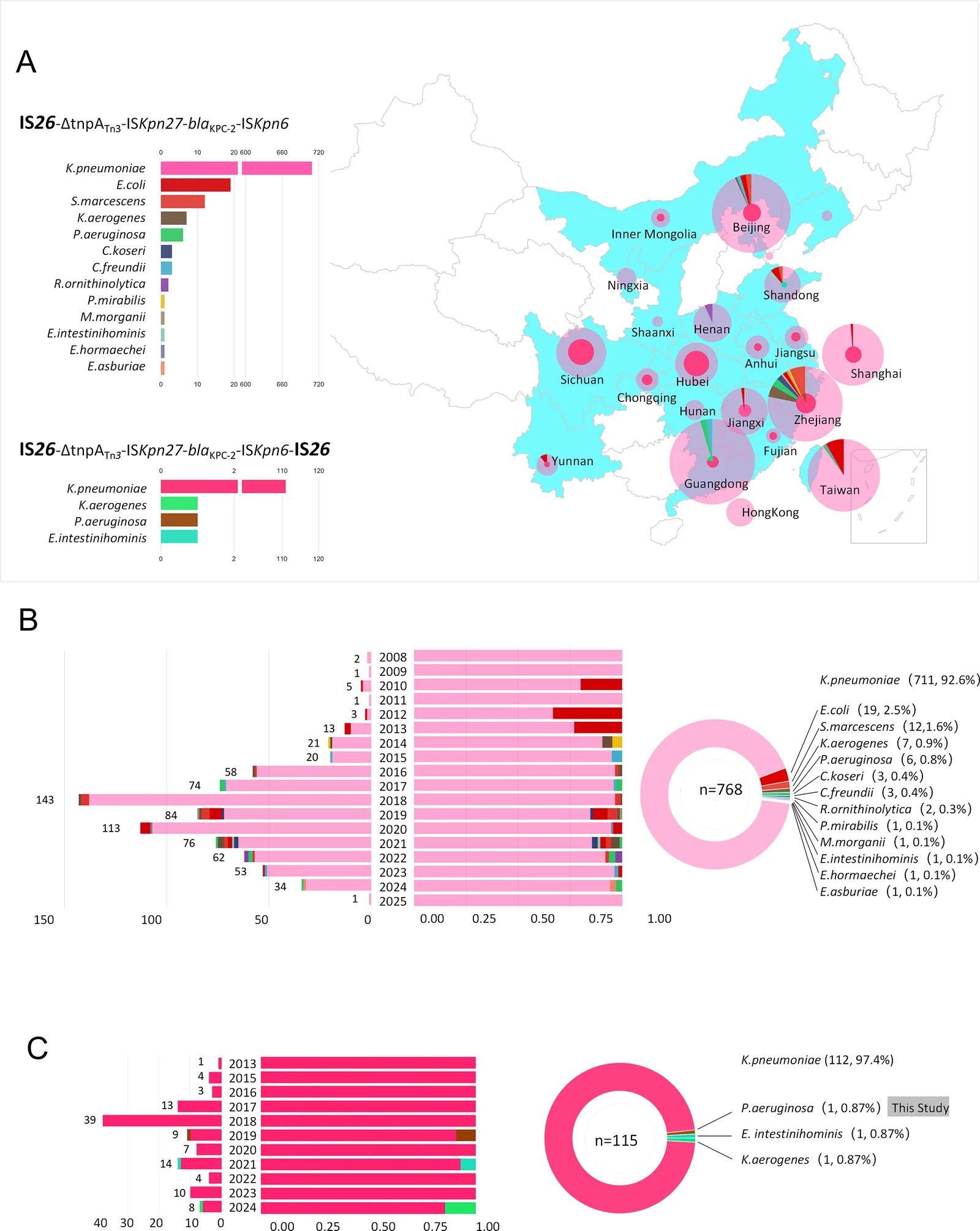
Geographical distribution, temporal trends, and population structure of 768 strains harboring the “IS*26-bla*_KPC_” structure and 115 strains carrying the “IS*26-bla*_KPC_-IS*26*” structure in China. (**A**) Geographical distribution of KPC-producing *P. aeruginosa* (KPC-PA) isolates across provinces in China. The diameter of each pie chart corresponds to the number of isolates, and different colors represent distinct host types. (**B**) Histogram showing the annual isolation frequency of 768 strains harboring the “IS*26-bla*_KPC_” structure, along with the relative frequency of different bacterial species. (**C**) Temporal distribution and relative proportion of 115 strains carrying the “IS*26-bla*_KPC_-IS*26*” structure (left panel), as well as the overall species distribution ratio of these strains (right panel).

For initial screening, a 4,746 bp fragment carrying the structure *IS26*-∆*tnpA_Tn3_-ISKpn27-bla_KPC-2_-ISKpn6* was analyzed via NCBI nucleotide BLAST; isolates were ranked by Max Score in descending order, and only those with Max Score >3,855, 100% query coverage, and 100% nucleotide identity were included to confirm the intact contiguous target structure (Fig. 1A and B). Globally, 799 qualified isolates were identified, with stark geographical inequality: 768 isolates were from China, far exceeding those from Sudan (11), the United States (9), Japan (3), the United Kingdom, India and South Korea (2 each), and Singapore and Canada (1 each). Due to the overwhelming predominance of Chinese isolates, subsequent analyses focused on the epidemiological features of this IS*26*-*bla_KPC_* structure in Chinese clinical populations. The 768 Chinese isolates covered 18 provinces, autonomous regions, and special administrative regions, spanning 13 common *Enterobacterales* species and *P. aeruginosa*, with obvious geographical clustering and species dominance. *K. pneumoniae* was the absolute dominant species, detected in all positive regions, with the highest counts in Beijing (122), Taiwan (100), and Zhejiang (93). Other species were sporadically distributed without regional epidemic trends, and Zhejiang showed the highest species diversity, harboring 8 additional bacterial species alongside *K. pneumoniae*. Notably, *P. aeruginosa* was only rarely detected in four regions, consistent with the distribution of the unique PA100 strain characterized in this study, confirming its low clinical detection rate and restricted distribution.

Subsequently, a second BLAST analysis was performed using a 5,450-bp fragment encompassing the extended genetic structure *IS26*-∆*tnpA_Tn3_-ISKpn27-bla_KPC-2_-ISKpn6-IS26*, targeting isolates carrying the full, contiguous extended structure (Fig. 1C). A total of 118 eligible isolates were identified globally: 2 from Canada, 1 from South Korea, all identified as *K. pneumoniae*, and 115 from China, which formed the focus of further detailed analysis. Among these 115 Chinese isolates, 112 were *K. pneumoniae*, 1 was *Enterobacter intestinihominis*, 1 was *K. aerogenes*, and 1 was *P. aeruginosa* (strain PA100), a novel isolate characterized exclusively in this study.

Detailed geographical and species distribution of these 115 Chinese isolates revealed further clustering: Sichuan Province accounted for 26 *K. pneumoniae* isolates, Hubei Province for 25 *K. pneumoniae* isolates, Zhejiang Province for 14 *K. pneumoniae* isolates plus 1 *K. aerogenes* isolate, Beijing for 12 *K. pneumoniae* isolates, Shanghai for 10 *K. pneumoniae* isolates, Chongqing and Guangdong Province for 4 *K. pneumoniae* isolates each, Jiangsu and Fujian Provinces for 3 *K. pneumoniae* isolates each, Anhui and Inner Mongolia for 2 isolates each, Yunnan Province for 1 *K. pneumoniae* isolate, Shandong Province for 1 *E. intestinihominis* isolate, and the novel *P. aeruginosa* PA100 isolate identified in Guangdong Province for this study.

We summarized the temporal trends and species composition of the 768 isolates harboring the one-flanking IS*26 bla_KPC_* structure. The histogram demonstrates a continuous upward trend in isolation frequency from 2008 to 2023, reaching a peak in 2018 (*n* = 143). The corresponding donut chart confirms strong species bias, with *K. pneumoniae* accounting for 92.6% (711/768) of all isolates, followed by *Escherichia coli* (19 isolates, 2.5%) and *Serratia marcescens* (12 isolates, 1.6%). We also presented the temporal dynamics and species distribution of the 115 isolates carrying the two-IS*26*-flanked *bla_KPC_* structure. Isolation frequency peaked in 2018 (*n*= 39), and *K. pneumoniae* represented 97.4% (112/115) of total isolates. Notably, *P. aeruginosa* accounted for only 0.87% (1/115) of these isolates, which is the PA100 strain characterized in this study, highlighting the extreme rarity of this extended two IS*26* flanked *bla_KPC_* structure in *P. aeruginosa* clinical isolates.

### Clinical and molecular characterization of coisolated *K. pneumoniae* KC122 and *P. aeruginosa* PA100

On 28 February 2024, a 90-year-old male patient in the intensive care unit of the Second Affiliated Hospital of Guangzhou Medical University was diagnosed with a concurrent infection involving *K. pneumoniae* KC122 and *P. aeruginosa* PA100. The patient had a history of radical cystectomy and urinary diversion for bladder cancer in 2015, with an indwelling urinary fistula postoperatively. Notably, a drug-susceptible *P. aeruginosa* isolate was detected from the same patient on 15 January 2024, but this strain was not available for further analysis. Whole-genome sequencing revealed the strain PA100 harbors a circular chromosome (GenBank accession: CP174573) and one plasmid (pPA100-1, GenBank accession: CP174574) and one circular transferable unit (pPA100-2, 4746 bp GenBank accession: CP174575).

**Fig 2:**
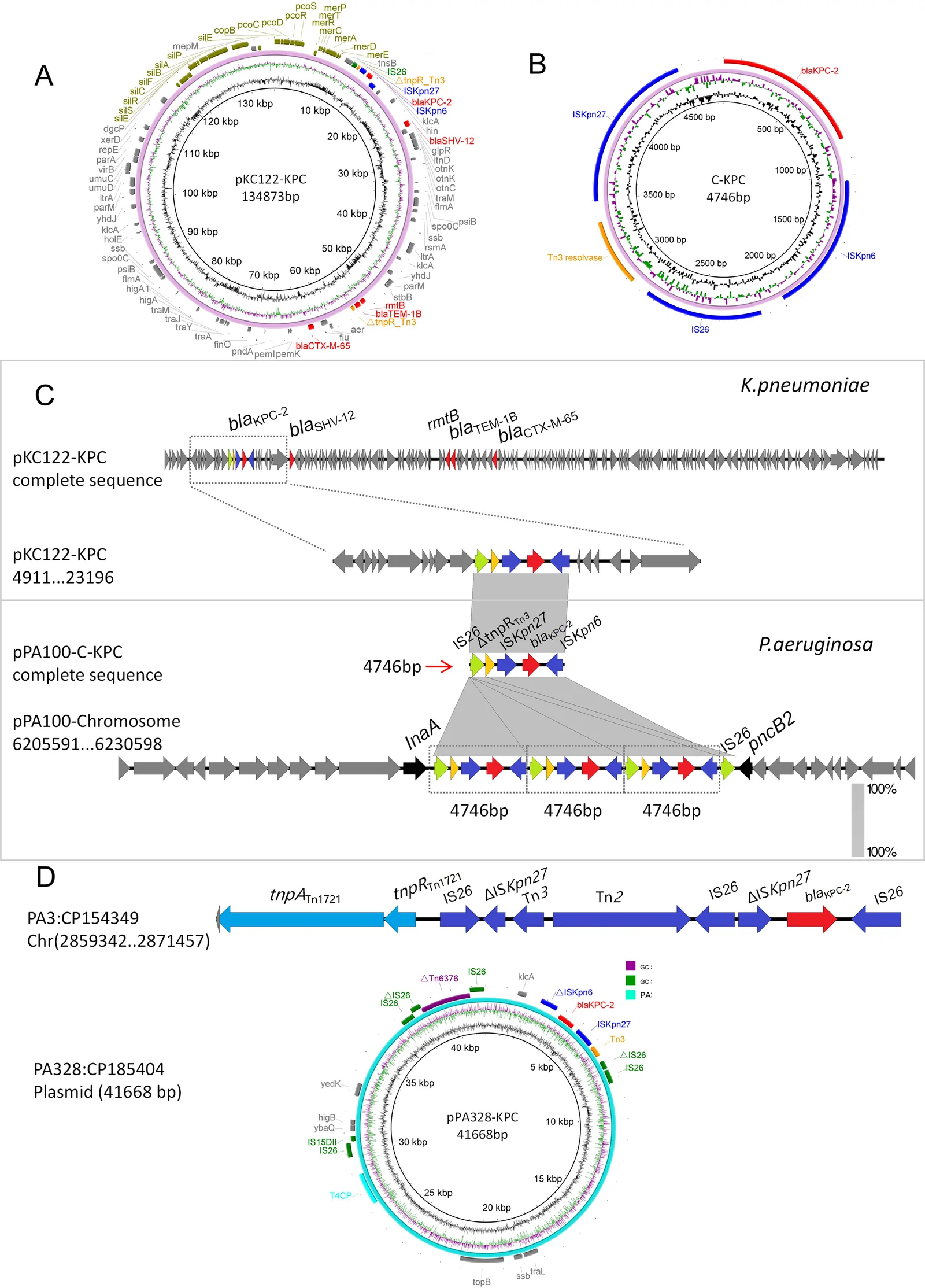
Schematic diagrams of *bla*_KPC_-associated genetic elements in relevant strains in this study. (**A**) Schematic map of the plasmid harboring *bla*_KPC_ in *K. pneumoniae* KC122 strain. (**B**) Schematic diagram of the 4,746 bp *bla*_KPC_ circular intermediate in *P. aeruginosa* PA100 strain. (**C**) Comparative schematic of *bla*_KPC_ genetic contexts: alignment of pKC122-KPC plasmid (positions 4,911 to 23,196), PA circular transposon (4,746 bp), and the corresponding chromosomal region of PA100 strain (positions 6,215,392 to 6,223,231). Gray shaded regions indicate 100% sequence homology between different chromosomal segments, and dashed lines denote the correspondence between chromosomal regions and the circular intermediate, with all genetic elements clearly annotated within the circular structure. (**D**) Genetic contexts of other two KPC-PA strains included in this study: PA3 strain carries *bla*_KPC_ via chromosomal Tn*1721* transposon insertion, while PA328 strain harbors *bla*_KPC_ via plasmid-borne Tn*6296*-like transposon integration. Red, orange-purple, blue, and gray arrows represent *bla*_KPC_ gene, transposition genes, insertion sequences, and other open reading frames (ORFs), respectively.

To elucidate the genetic architecture of IS*26*-associated *bla_KPC_* contexts, we performed complete sequencing and comparative genomic analysis of representative plasmids and chromosomal loci from KC122 and PA100. Plasmid pKC122-KPC (134,873 bp) from KC122 is a large non-conjugative plasmid harboring multiple antimicrobial resistance determinants, including *bla_KPC-2_*, *bla_SHV-12_*, *bla_TEM-1B_*, and *bla_CTX-M-65_*, alongside heavy metal resistance operons (*mer*, *sil*) (Fig. 2A). Notably, oriTfinder analysis indicated that pKC122-KPC lacks an identifiable oriT, relaxase, T4CP, and T4SS, suggesting that this plasmid is unlikely to undergo autonomous conjugative transfer (Fig. 2A). The *bla_KPC-2_ of* KC122 was found in one-IS*26*-flanked *bla*_KPC_ cassette (IS*26*-∆*tnpA*_Tn3_-*ISKpn27-bla_KPC-2_-ISKpn6*), which represents the predominant *bla*_KPC-2_ genetic context among clinical *K. pneumoniae* isolates (Fig. 1). Here, Δ*tnpR* indicates a truncated *tnpR* gene within the Tn*3* transposon module. No tandem repeats of *bla*_KPC_ region or circular transposition intermediates were observed (data not shown).

In PA100, the IS*26*-bracketed *bla_KPC-2_* structure (*IS26*-∆*tnpA*_Tn3_-*ISKpn27-bla_KPC-2_- ISKpn6-IS26*) is chromosomally integrated at the *InaA* locus adjacent to the *pncB2* gene, with the capacity to form multiple tandem repeat arrays mediated by IS*26*, representing a rare or previously unreported configuration in *P. aeruginosa* (Fig. 2B and C). Furthermore, we identified a circular 4,746-bp *bla_KPC-2_* cassette corresponding to the circular transferable unit. This circular intermediate is proposed to be generated via IS*26*-mediated homologous recombination from the parental double-IS*26*-flanked chromosomal structure, representing a key intermediate in the horizontal dissemination of this carbapenem resistance module. Comparative genomic analysis uncovered *bla*_KPC-2_ genetic contexts with 100% nucleotide identity of the core *bla*_KPC-2_ cassettes PA100 and KC122 is highly suggestive of horizontal gene transfer of this resistance module between *Enterobacterales* and *P. aeruginosa*.

Additionally, we present the genetic contexts of two *P. aeruginosa* reference strains used in subsequent phenotypic assays—PA3 (CP154349) and PA328 (CP185404), both belonging to sequence type ST463. These two strains harbor *bla_KPC-2_* contexts that differ in their genetic location (chromosomal vs plasmid-borne): PA3 carries a chromosomally integrated *bla_KPC-2_* cassette within a Tn*1721*-derived transposon flanked by *IS26* and a truncated *ISKpn27*; in contrast, PA328 carries *bla_KPC-2_* on a 41,668-bp plasmid (pPA328-KPC), embedded within a Tn*6376* (Tn*6296*-like) transposon flanked by ∆*ISKpn6* and *ISKpn27* (Fig. 2D).

### Validation of the *bla_KPC_* genetic context and circular transposition intermediate in PA100

**Fig 3:**
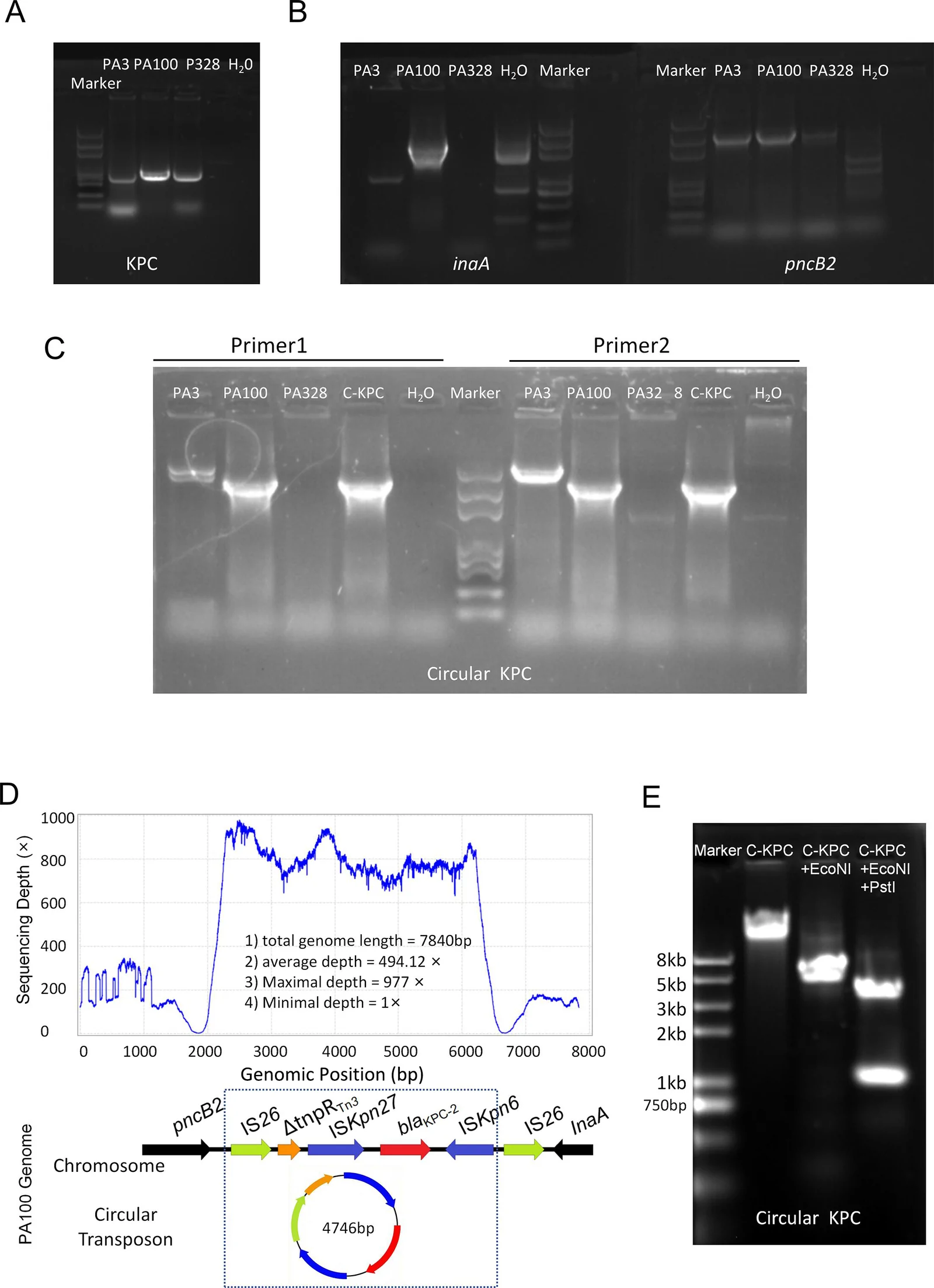
Identification of the *bla*_KPC_ circular transposon structure in *P. aeruginosa* PA100 strain. (**A**) PCR identification of the *bla*_KPC_ gene in KPC-PA strains. (**B**) PCR verification of the genetic context harboring the “IS*26-bla*_KPC_-IS*26*” structure in PA100 strain. (**C**) Validation of the circular *bla*_KPC_ structure in KPC-PA strains using two pairs of specific primers via PCR. (**D**) WGS read depth profile aligned with the genetic structure map of PA100 strain, showing sequencing depth (vertical axis, ×) across a ~7,840 bp genomic region (horizontal axis, bp). (**E**) Agarose gel electrophoresis of the circular *bla*_KPC_ structure (C-KPC) extracted using a plasmid extraction kit: Lane 1: DNA Marker; Lane 2: Native C-KPC; Lane 3: Linearized C-KPC digested with *Eco*NI; Lane 4: C-KPC double-digested with *Eco*NI and *Pst*I (Note: 5 μg of DNA was used for digestion, and the exposure intensity and exposure time were increased).

We first present the electrophoretogram of *bla_KPC_* detection using specific PCR primers (Fig. 3A). Based on WGS data, we further designed specific PCR primers targeting the flanking genes of the *IS26-bla_KPC_-IS26* structure, which verified the accuracy of the *InaA* and *pncB2* loci adjacent to the resistance module in the assembled genome (Fig. 3B). This composite genetic structure was unique to PA100 compared with reference strains PA3 and PA328. To confirm the circular structure, two pairs of outward-facing specific primers targeting *bla_KPC_* and its flanking regions were designed for long-range PCR; amplicons were only generated if the circular intermediate existed (Fig. 3C). WGS assembly revealed a distinct circular intermediate of 4,746 bp, harboring the *bla_KPC-2_* gene and flanking *IS26* elements. The circular unit corresponds to GenBank accession CP174575 and is annotated as IS*26*-∆*tnpA*_Tn3_-*ISKpn27-bla_KPC-2_- ISKpn6*; the head-to-tail junction was verified by inverse PCR, restriction enzyme digestion, and Sanger sequencing of the amplicon. Read-depth analysis from WGS indicated approximately four tandem copies of this 4,746-bp fragment on the chromosome. This circular intermediate is proposed to be formed via *IS26*-mediated homologous recombination, providing a molecular basis for the horizontal dissemination of *bla_KPC_* (Fig. 3D).

We performed nucleic acid extraction from PA100 using a plasmid extraction kit and achieved concentrated preparation of this circular transposition intermediate through extensive optimization. The sequence of the extracted circular element was confirmed by Sanger sequencing (data not shown), and its structure was further validated by restriction enzyme digestion: linearization with *Eco*NI and double digestion with *Eco*NI and *Pst*I yielding expected fragments of 1,047 bp and 3,704 bp (Fig. 3E).

Serial passaging of PA100 for more than 10 consecutive generations consistently yielded the same circular transposition intermediate (gel images not shown). Collectively, these multiple lines of experimental evidence confirm the stable presence and precise molecular structure of the *bla_KPC_*-bearing circular intermediate in strain PA100.

### Functional validation of the circular *bla*_KPC_ transposition in PA100

**Fig 4:**
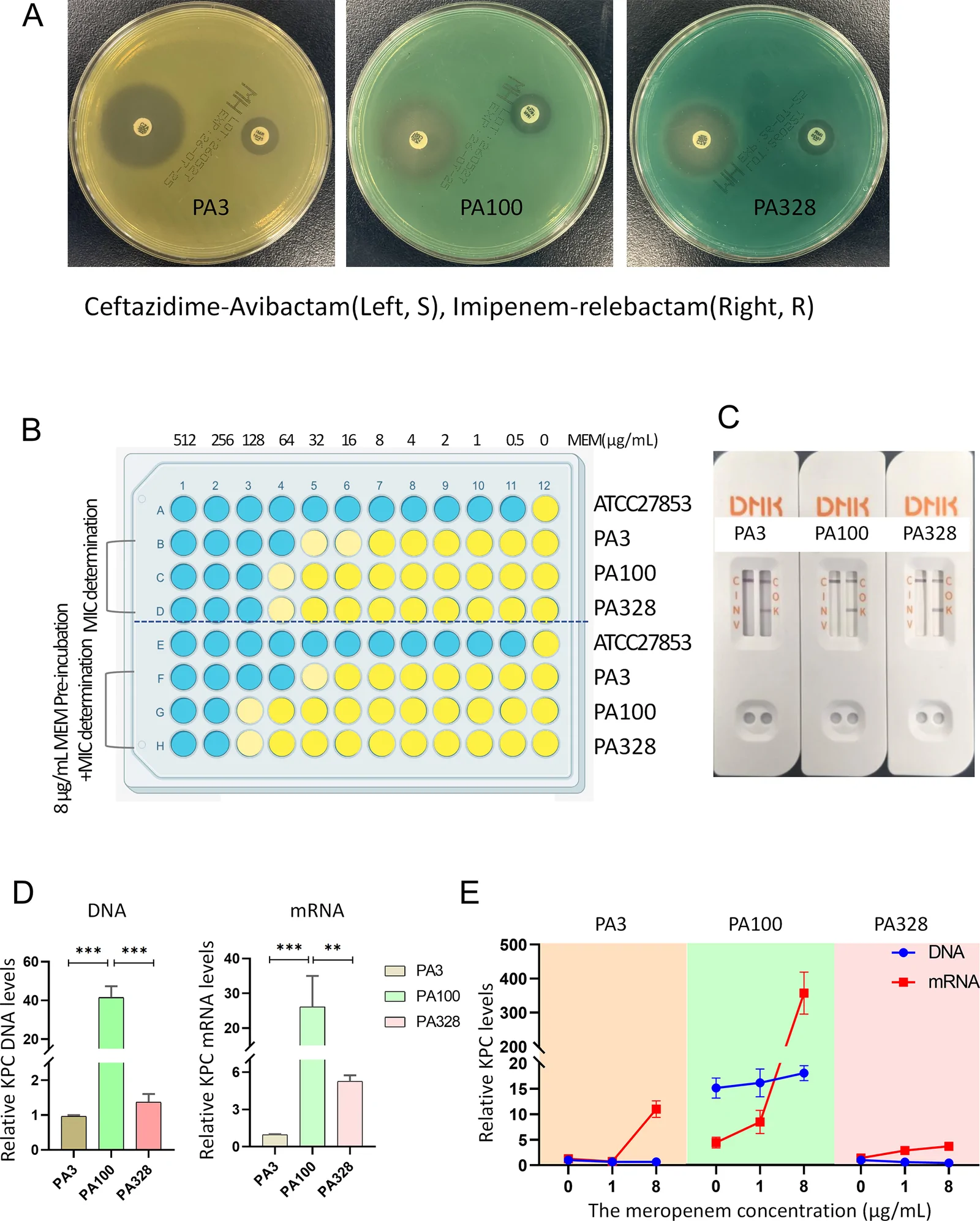
*bla*_KPC_ DNA copy numbers, mRNA expression levels, and drug susceptibility profiles of PA100 strain harboring *bla*_KPC_ circular transposon, with PA3 (chromosomal *bla*_KPC_) and PA328 (plasmid-borne *bla*_KPC_) as control strains. (**A**) Kirby-Bauer (KB) disk diffusion assay results of PA3, PA100, and PA328 strains against ceftazidime-avibactam. (**B**) Minimum inhibitory concentration (MIC) determinations of PA3, PA100, and PA328 strains, as well as MIC values after pretreatment with 8 μg/mL meropenem. (**C**) Detection of carbapenemase expression in KPC-PA strains (PA3, PA100, PA328) via immunochromatographic assay, with strain-specific carbapenemase expression determined by the presence of control line (C) and test line (T) bands. (**D**) Relative DNA copy numbers and mRNA expression levels of *bla*_KPC_ in three *P. aeruginosa* strains (PA3, PA100, PA328) with different *bla*_KPC_ carriage patterns, presented as bar graphs. Values are shown as mean ± SD with three independent replicates per group. Two-group comparisons were conducted by Student’s *t*-test. Statistical significance thresholds: **P < 0.01 and ***P < 0.001. (**E**) Line graphs show fold changes in *bla*_KPC_ DNA copy numbers and mRNA expression of PA3, PA100, and PA328 cultured with meropenem at 0, 1, and 8 μg/mL. Values represent mean ± SD (*n* = 3 biological replicates per group).

Antimicrobial susceptibility testing was conducted for the target strain PA100, with PA3 and PA328 as reference strains (Table 1). PA100 displayed an extensively drug-resistant phenotype, with a multidrug resistance profile largely consistent with the two reference strains. All three strains were uniformly resistant to all tested β-lactams, including cephalosporins, aztreonam, and β-lactam/β-lactamase inhibitor combinations, as well as high-level carbapenem resistance consistent with *bla_KPC_* production. Notably, PA100 and PA328 showed identical carbapenem MICs (meropenem 64 μg/mL; imipenem 64 μg/mL), while PA3 had slightly lower but resistant MICs (meropenem/imipenem 32 μg/mL) (Table 1). All strains remained sensitive to ceftazidime-avibactam, with inhibition zones of 24.5 mm (PA100), 22 mm (PA328), and 33 mm (PA3) (Fig. 4A). The MIC values of ceftazidime-avibactam against these three strains were 4/1 μg/mL (PA100), 4/1 μg/mL (PA328), and 1/0.25 μg/mL (PA3), respectively, all interpreted as susceptible (S). Consistently, all isolates were susceptible to cefiderocol. PA100 and the *bla*_KPC_-plasmid-carrying strain PA328 shared identical cefiderocol MICs, which exceeded the MIC measured for chromosomally *bla*_KPC_-harboring PA3 (Table 1). ResFinder analysis revealed that PA100 carried six chromosomal copies plus one additional copy on a circular intermediate, whereas PA3 and PA328 each contained a single chromosomal or plasmid-borne copy, respectively (Table 2).

**TABLE 2:** Key acquired resistance genes identified in KPC-PA isolates by ResFinder^*a*^

Isolate	*bla*_KPC-2_ (copy number/location)	Other acquired ARGs	Notes
PA100	6× chromosome + 1× circular intermediate	*aph(3')-IIb*, *fosA*, *catB7*, *crpP*, *tmexD2*	Chromosomal *bla*_PAO_, *bla*_OXA-50_ also present
PA3	1× chromosome	*aph(3')-IIb*, *aac(6')-IIa*, *ant(2'')-Ia*, *bla*_CARB-2_, *bla*_OXA-486_, *fosA*, *catB7*, *crpP*, *sul1*	Chromosomal *bla*_PAO_ also present
PA328	1× plasmid	*fosA*, *catB7*, *crpP*, *bla*_OXA-486_, *bla*_PDC-374_	Lacks *aph(3')-IIb* and *bla*_PAO_

^*a*^ Coverage ≥98.1% and identity ≥96.5% for all listed genes. Chromosomal *bla*_PAO_, *bla*_OXA-50_/*bla*_OXA-486_, and *bla*_PDC_ are intrinsic β‑lactamases of *P. aeruginosa* and were not considered acquired resistance determinants.

Although both PA100 and PA3 carry *bla*_KPC_ on their chromosomes, the antimicrobial susceptibility profile of PA100 (which harbors chromosomally encoded *bla*_KPC_) is inconsistent with that of PA3 (carrying chromosomally encoded *bla*_KPC_ within Tn*1721*) but is more closely aligned with that of PA328 (carrying *bla*_KPC_ on a plasmid). The MIC of meropenem against PA100 was determined via broth microdilution assay, yielding a baseline MIC of 64 μg/mL. Following pre-treatment with 8 μg/mL meropenem for 6 h, the MIC of PA100 to meropenem increased to 128 μg/mL (Fig. 4B). The expression of KPC enzyme in PA100 was detected using a carbapenemase detection kit (colloidal gold immunochromatography), yielding a positive result (Fig. 4C). Next, we performed qPCR to determine the relative DNA and mRNA levels of the *bla_KPC_* transposition intermediate in PA100, alongside PA3 and PA328 as reference controls. The DNA copy number of the *bla_KPC_* intermediate in PA100 was approximately 40-fold higher than that in PA3, and the relative mRNA expression level was roughly 25-fold higher compared with PA3 (Fig. 4D). We subsequently assessed *bla_KPC_* DNA and mRNA expression levels following meropenem induction. Upon exposure to 8 μg/mL meropenem for 6 h, the relative mRNA level of *bla_KPC_* in PA100 increased by nearly 70-fold relative to the uninduced group, a markedly higher upregulation fold than that observed in PA3 (approximately 10-fold) and PA328 (approximately 5-fold) (Fig. 4E).

Conjugation assays were conducted using recipient strains J53 and rifampicin-resistant PAO1 (PAO1^Rif^) to evaluate the horizontal transfer capacity of the *bla*_KPC_ resistance element, yet all transfer experiments yielded negative results. Transformation assays were further performed with purified circular intermediate DNA, encompassing heat-shock transformation into *E. coli* DH5α and electroporation into *P. aeruginosa* PAO1. A synthetic high-copy plasmid harboring *bla*_KPC_ was used as the positive control, and all assays were carried out in biological triplicate. No positive transformants were recovered after selective incubation. Given the extremely low inherent frequency of transposition events under laboratory conditions, dedicated transposition validation assays were not performed in this study.

### Characterization of PA100-derived OMVs and *bla_KPC_* detection

**Fig 5:**
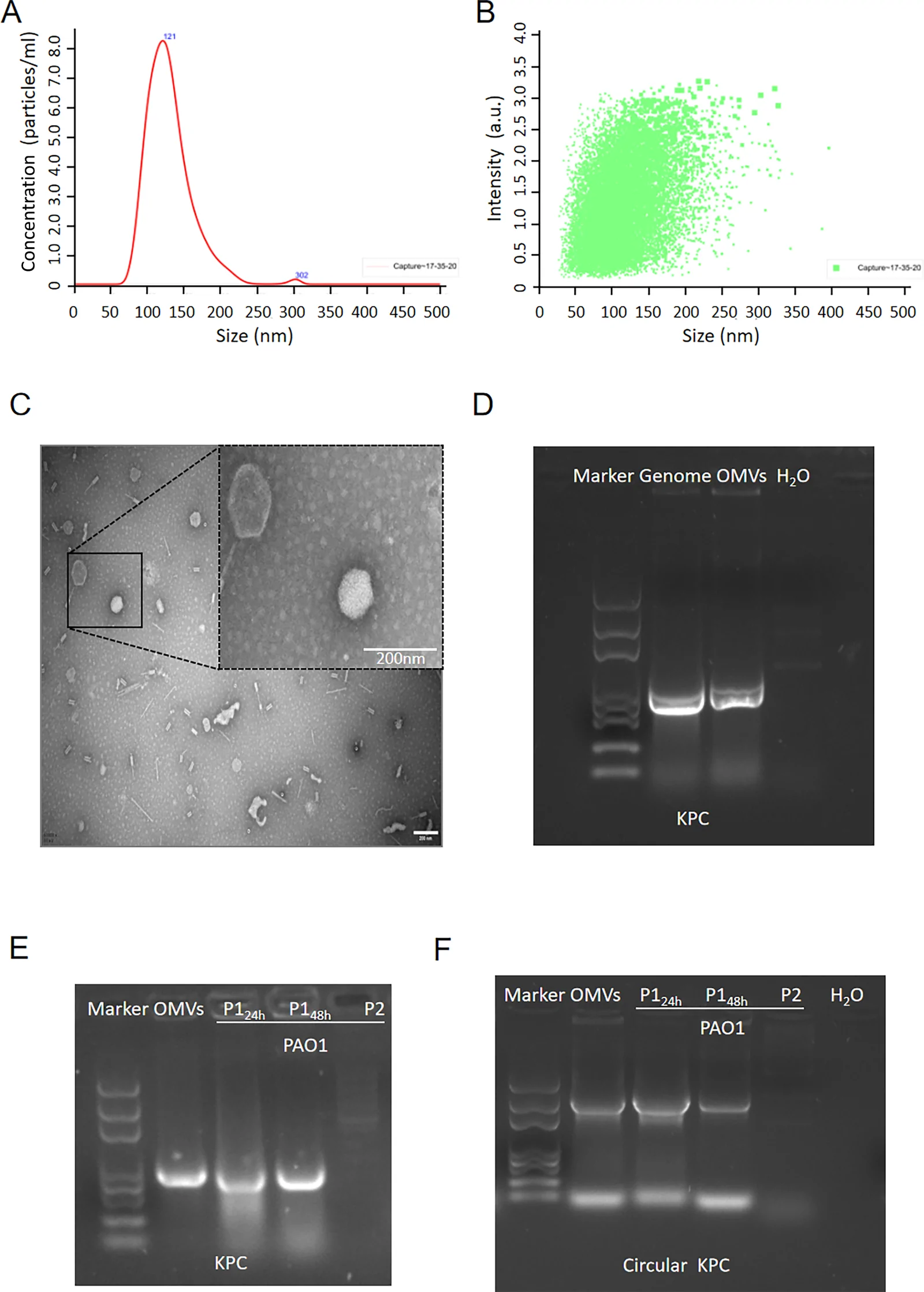
Characterization of PA100-derived OMVs and their mediation of *bla*_KPC_ horizontal transfer to PAO1 strain. (**A and B**) Particle size-concentration distribution of purified OMVs from PA100 analyzed by averaged total internal reflection fluorescence lifetime imaging (FTLA). The horizontal axis represents particle size (nm), and the vertical axis represents particle concentration (particles/ml), showing a main peak at 121 nm. (**C**) TEM image of PA100-derived OMVs, displaying typical vesicle structures with the target size. (**D**) PCR amplification and agarose gel electrophoresis analysis of the *bla*_KPC_ gene in PA100 OMVs. Lane 1: DNA Marker; Lane 2: PA100 genomic DNA (positive control); Lane 3: Purified OMVs; Lane 4: Nuclease-free H₂O (negative control). (**E**) Detection of the *bla*_KPC_ gene in PAO1^Rif^ after co-culture with PA100-derived OMVs. Lane 1: DNA Marker; Lane 2: PA100-derived OMVs (positive control); Lanes 3–5: PAO1 single colony at 24 h and 48 h post-coincubation (Passage 1) and Passage 2 (P2) after isolation. (**F**) Detection of the circular *bla*_KPC_ structure by inverse PCR in PAO1ᴿⁱᶠ after co-cultured with OMVs. Lane 1: DNA Marker; Lane 2: PA100-derived OMVs (positive control); Lanes 3–5: PAO1 single colony at 24 h and 48 h post-coincubation (P1) and Passage 2 (P2) after isolation. Lane 6: Nuclease-free H₂O (negative control).

We subsequently isolated OMVs from PA100 and performed comprehensive characterization of the purified vesicles via nanoparticle size distribution analysis, TEM, and molecular detection of *bla_KPC_* within the vesicle cargo. As shown in Fig. 5A and B, size distribution analysis of the purified OMVs revealed a single predominant peak at 121 nm with a minor secondary peak at 302 nm, confirming the isolated particles fall within the typical nanoscale size range of OMVs; the intensity-size scatter plot further verified the high homogeneity of the OMVs, with the majority of particles concentrated in the 50–200 nm range, consistent with the size distribution profile. TEM imaging displayed intact, spherical vesicle structures with diameters consistent with the nanoparticle tracking analysis results, confirming the successful isolation of morphologically intact and viable OMVs (Fig. 5C). Furthermore, agarose gel electrophoresis of specific PCR products confirmed the presence of *bla_KPC_* within the purified OMVs: a distinct target band corresponding to the *bla_KPC_* gene was detected in the OMVs sample lane, alongside clear bands in the positive control (genomic DNA) lane and no band in the negative control (ddH₂O) lane (Fig. 5D). According to our experimental enumeration, the transfer frequency was approximately 5 × 10^−9^ transformants per recipient cell. These findings support that the *bla_KPC_*-carrying circular transposon is packaged and delivered via PA100-derived outer membrane vesicles.

PA100-OMVs were co-incubated with rifampicin-resistant PAO1 via the conventional co-culture method for 6 h, after which the co-culture system was serially diluted, spread on both meropenem and rifampicin-containing LB agar plates, and dual-resistant colonies were observed following 24–48 h of incubation at 37℃. PCR amplification was performed to verify *bla_KPC_* gene presence and PAO1 identity in the isolated resistant colonies: primers targeting *bla_KPC_* yielded expected-size positive amplification products (Fig. 5E), while inverse primers specific to *bla_KPC_* also detected positive signals, confirming the presence of circular *bla_KPC_* in the isolated PAO1^Rif^ strains. Notably, *bla_KPC_* was not detectable in Passage 2 (Fig. 5F). These results indicated that *bla_KPC_*-carried OMVs could be transferred to PAO1, but the recipient PAO1 failed to maintain detectable *bla*_KPC_ after Passage 2, suggesting that stable chromosomal integration was not demonstrated under the current assay conditions. Accordingly, the OMV experiment supports transient gene delivery rather than stable inheritance in recipient PAO1^Rif^.

## DISCUSSION

The global spread of CRPA threatens clinical antimicrobial therapy, with *bla_KPC_* being a key carbapenemase gene mediating broad β-lactam resistance (7, 31, 32). This study identifies and characterizes a novel IS*26*-mediated circular *bla*_KPC-2_ transposon intermediate in the rarely reported clinical ST2483 *P. aeruginosa* strain PA100, mapped to the chromosomal *InaA*/*pncB2* intergenic region with a unique multicopy tandem arrangement of *bla*_KPC-2_. We show that this circular element can be detected in PA100-derived OMVs and transiently detected in PAO1Rif after OMV exposure and delineate its association with the isolate’s antimicrobial resistance phenotype and *bla*_KPC-2_ expression regulation.

**Fig 6:**
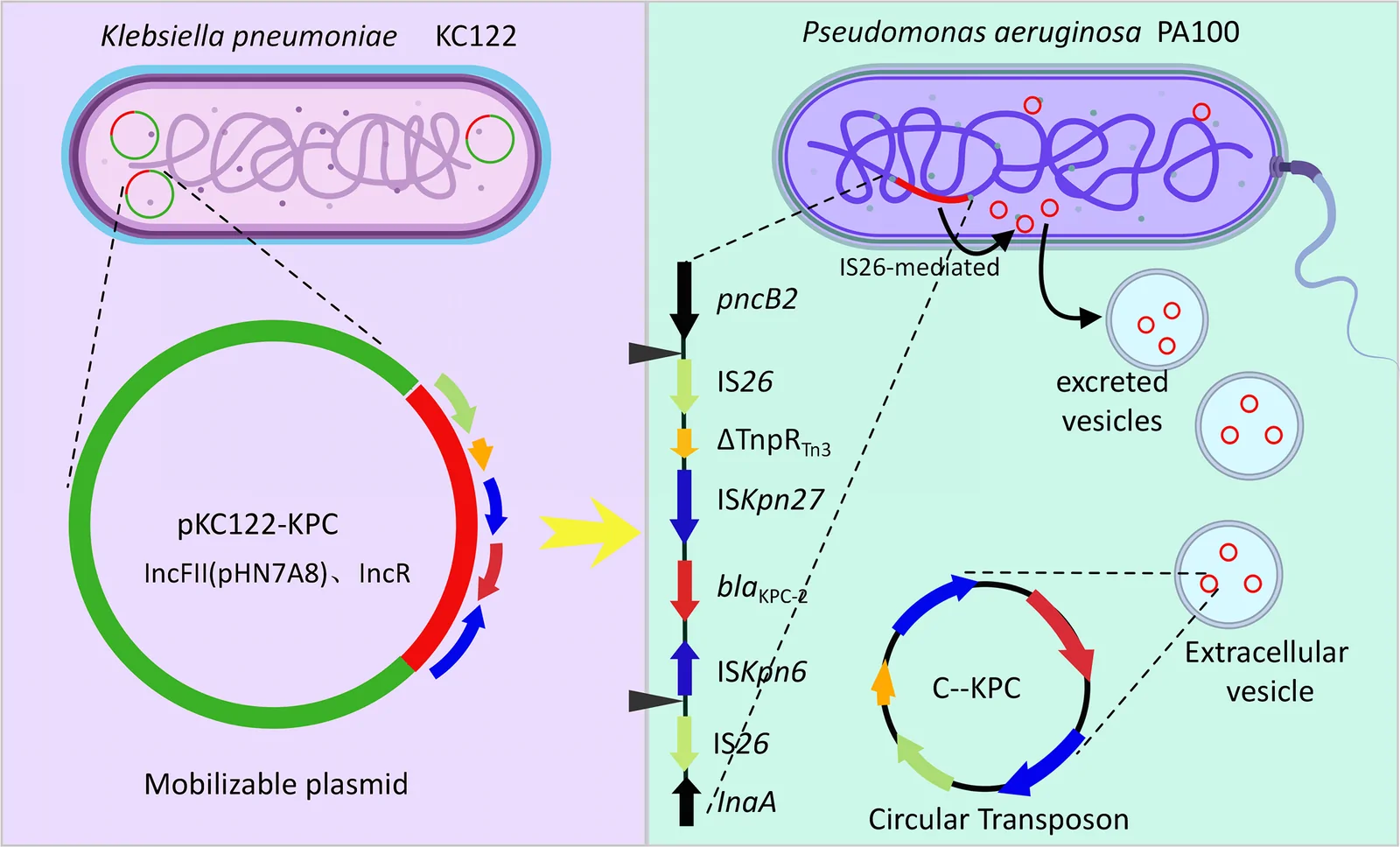
Proposed horizontal dissemination pathways of *bla*_KPC_ mediated by plasmids and circular transposons.

IS*26* is a pivotal mobile genetic element driving resistance gene mobilization in Gram-negative bacteria via homologous recombination (14, 15), and its role in *bla*_KPC_ dissemination and evolution was further clarified in this study. BLAST analysis against the NCBI database showed that the double-IS*26*-flanked *bla*_KPC_ structure was predominantly present in *K. pneumoniae*, extremely rare in other bacterial species, and unreported in *P. aeruginosa*. Notably, the plasmid pKC122-KPC from *K. pneumoniae* strain KC122 (co-infected with PA100) carries a *bla_KPC-2_* genetic environment 100% identical to that of strain PA100 (Fig. 6). Since pKC122-KPC is non-conjugative and cannot mediate independent horizontal transfer, IS*26* is speculated to mediate *bla*_KPC_ transfer between bacteria or to have contributed to the remodeling of a shared *bla*_KPC_ module after acquisition. Alternatively, PA100’s *bla*_KPC_ may derive from other KPC-producing *K. pneumoniae* strains, with IS*26* facilitating its acquisition. Serial passaging, inverse PCR, and restriction enzyme digestion confirmed stable maintenance of the circular *bla*_KPC_ transposon in PA100, with IS*26*-mediated intramolecular homologous recombination proposed to underlie the formation of the circular transferable unit. Unlike the classic Tn*1721* and Tn*6376* transposon backbones associated with *bla*_KPC_ in ST463 isolates, IS*26*-bracketed *bla*_KPC-2_ in strain PA100 existed as a multicopy tandem array and was associated with higher *bla*_KPC-2_ copy number, higher transcriptional response to meropenem, and high-level carbapenem resistance. This suggests IS*26* not only mediates *bla*_KPC_ mobilization but also enhances its resistance phenotype, deepening our understanding of IS*26*’s role in *bla*_KPC_ evolution.

Previous studies have predominantly focused on *bla*_KPC_ genes carried by plasmids or classical linear transposons in *Enterobacterales* (33–35) and common *P. aeruginosa* sequence types (6, 8, 13, 21), with limited reports describing IS*26*-mediated circular transposition intermediates or OMV-mediated transmission in *P. aeruginosa*—leaving a gap in understanding CRPA dissemination. As OMVs are increasingly recognized as key mediators of non-classical horizontal gene transfer in Gram-negative bacteria (36, 37), our study confirmed that PA100 secretes intact, typical *P. aeruginosa* OMVs, which can encapsulate the full-length circular *bla*_KPC-2_ transposon, potentially protecting it from extracellular nuclease degradation and facilitating its extracellular dissemination of this resistance element. Negative conjugation and transformation assays further demonstrate that *bla*_KPC_ does not rely on classical horizontal gene transfer, indicating OMVs as an alternative, non-plasmid-dependent transmission route—effectively filling the existing gap in CRPA resistance dissemination research and highlighting the novelty of our findings. Notably, we did not detect the persistent presence of transposon structures in recipient bacteria, which is presumably attributed to the low probability of transposon integration into the chromosome, leading to its undetection in the current study.

Despite the novel findings on IS*26*-mediated circular *bla*_KPC-2_ transposon formation and OMV-mediated transmission in *P. aeruginosa* PA100, this study has several limitations. First, the focus on only PA100 (ST2483) and KC122 limits the generalizability of findings. Second, the molecular mechanisms of OMV encapsulation, IS*26*-mediated recombination, and OMV secretion regulation remain unclear; the present data do not determine whether tandem formation depends only on IS*26*-mediated homologous recombination, replication-associated events, local sequence architecture at the *inaA-pncB2* intergenic region, or other host factors. Third, persistent transposon structures in recipient bacteria were not detected, with no direct evidence of integration efficiency. Additionally, *in vivo* transmission efficiency and clinical impact of OMV-mediated transposon transfer were not verified. Nevertheless, the role of these *oprD* alleles and efflux-related variants in carbapenem resistance was not functionally characterized in this study.

In summary, this study clarifies the critical roles of IS*26* and OMVs in *bla*_KPC_ dissemination and evolution in *P. aeruginosa*. Notably, PA100 belongs to the rarely isolated ST2483 *P. aeruginosa* clone, which carries a transmissible circular *bla*_KPC_ transposon mediated by OMVs—posing potential risks of nosocomial outbreaks given its unusual prevalence and resistance transmission capacity. We confirmed IS*26* mediates circular *bla*_KPC_ transposon formation in PA100, which are encapsulated in OMVs for stable, non-classical, non-plasmid-dependent transfer—filling the gap in CRPA resistance research previously focused on plasmids or linear transposons (Fig. 6).

## Data Availability

The genome sequence of P. aeruginosa PA100, which contained a chromosome, one plasmid, and one circular transferable unit, was submitted to GenBank under the accession numbers: CP174573-CP174575. The chromosome sequence carrying the bla_KPC_ gene from strain PA3 is available under accession number CP154349, and the plasmid sequence harbouring bla_KPC_ from strain PA328 is assigned accession number CP185404.

## SUPPLEMENTAL MATERIAL

Supplemental tables aac.00582-26-s0001.xlsx Tables S1 to S3.

ASM does not own the copyrights to Supplemental Material that may be linked to, or accessed through, an article. The authors have granted ASM a non-exclusive, world-wide license to publish the Supplemental Material files. Please contact the corresponding author directly for reuse.
